# Migratory Shorebird Gut Microbes are not Associated with Bivalve Prey in Monsoon Tropical Australia

**DOI:** 10.1007/s00284-024-03628-6

**Published:** 2024-03-12

**Authors:** Chava L. Weitzman, Zarah Tinning, Kimberley A. Day, Stephen T. Garnett, Keith Christian, Karen Gibb

**Affiliations:** Research Institute for the Environment and Livelihoods, Charles Darwin University, Brinkin, NT Australia

## Abstract

Migratory animals can carry symbionts over long distances. While well-studied for parasite and pathogen transmission, less is known about use of this route by other symbiotic taxa, particularly those non-pathogenic. Here we ask the question of whether gut bacteria can be spread between continents by long-distance bird migration, although gut microbiomes in birds may not be as stable or persistent as those of non-volant animals. We used amplicon sequencing of both bacterial 16S rRNA gene and *Vibrio*-centric hsp60 gene to determine whether the faecal bacteria of migratory great knots (*Calidris tenuirostris*) also occur in their main food source in Northern Australia or in nearby sand, comparing samples before and after the birds’ long-distance migration. Our data suggest that there is little connectivity among the bacterial microbiomes, except in the bivalve prey. Our results are consistent with previous studies finding that bird faecal microbiomes were not host-specific and contrast with those showing an influence of diet on bird faecal bacteria. We also found little connectivity among *Vibrio* spp. However, although faecal sample sizes were small, the dominance of different individual *Vibrio* spp. suggests that they may have been well-established in knot guts and thus capable of moving with them on migration. We suggest that the physiological impacts of a long-distance migration may have caused shifts in the phyla comprising great knot faecal communities.

## Introduction

Faecal microbiomes contain a combination of bacteria recently ingested with food and long-term colonisers of the animal gut. As faeces contain living bacteria, hosts may provide transport for microbial spread [1], and animals that move long distances may consequently spread symbionts far and wide. For example, migratory birds are responsible for the long-distance movement of multiple pathogens and parasites, gastro-intestinal and otherwise [2]. Similarities between bird and bat microbiomes suggest that requirements to reduce weight to improve flight efficiency may extend to a reduction in gastro-intestinal microbial biomass [3]. In contrast to non-flying mammals, birds have less reliance on gut microbes for nutrient acquisition [4]. Possibly as a consequence, the gut microbial communities in birds have a reduced host-phylogenetic signal, lower than those in non-flying mammals and many reptiles [3, 5, 6]. Though many studies identify diet as a driving force of gut microbiomes [7], the extent to which diet type shapes the gut communities of birds appears to be weak [5, 8, 9], with an indication that at least some and possibly most faecal microbes may represent transient, foodborne taxa [10, 11]. Consequently, we lack a strong understanding of whether migratory birds have the capacity to spread dietary bacteria, particularly those not associated with disease in the birds, across long distances.

A total of 34 species of migratory shorebirds using the East Asian-Australasian Flyway regularly reach Australia, with great knots (*Calidris tenuirostris*), one of the more abundant species, having been tracked from Northern Australia to their breeding grounds in Siberia [12]. We focused this study on great knots not just for their long migration, but also because they gather in large flocks at our study site, facilitating identification and recovery of their faeces. Here, we assessed the bacterial connectivity in a great knot trophic interaction at two times of year—before and after birds had completed a long-distance migration. To detect connectivity among samples and sample types, we therefore use network analysis alongside other diversity analyses because differences in bird faecal bacteria before and after migration could be associated with multiple factors. For example, seasonal changes in ingested bacteria (from their bivalve food source), a change in resident microbes because different foods are eaten during migration, or physiological changes associated with migration could all impact bird microbiomes. Throughout their large migratory range, great knots consume a varied diet, from predominantly small bivalves at our study site in Northern Australia, to different local invertebrates at stopover points in China, and even supplementing their invertebrate diet with berries and nuts at their breeding grounds [13–15]. Alongside whole bacterial microbiome data, we focused on detecting *Vibrio* species in the samples. Bacteria in the genus *Vibrio* include pathogenic and non-pathogenic taxa associated with human and wildlife diseases, and previous studies suggest that birds ingest potentially pathogenic vibrios and may spread them long distances [16, 17].

We aimed to identify the bacterial connectivity between great knots, their main food source, and the environment, to address three main hypotheses. First, we hypothesised that, while the three community types (bird faeces, bivalve, sand) would have different community composition, they would exhibit connectivity in network analysis, suggesting movement of microbes through the system. Second, we hypothesised that the connectivity between faecal and bivalve samples would be greater among samples collected before the birds’ migration, when they had been feeding vigorously to build energy reserves, than when they had just returned and so had little opportunity to feed on local bivalves. Differences in connectivity before and after migration would then indicate the extent to which birds retain some bacteria for extended periods of time (particularly if due to an infection). Third, we hypothesised that naturally occurring, potentially pathogenic, *Vibrio* species would be detectable in the bird faecal samples, suggesting a capacity for birds to move potential pathogens over long distances.

## Materials and Methods

We collected samples of great knot faeces, bivalves (the birds’ main food source), and nearby sand near Buffalo Creek, NT (12° 20′ 13.56″ S, 130° 54′ 30.73″ E) in March 2022 immediately before the birds’ long-distance migration to their Palearctic breeding grounds, and again in September 2022 immediately after they had returned (*n* = 8 samples per type per sampling). At this site, great knots predominantly consume small pipi bivalves (*Paphies altenai*), which occur just below the surface of the sand and are the most abundant intertidal invertebrate species [13]. Great knots feed in flocks near the edge of the sea during low tide, and flock sizes during our sampling were hundreds to > 1000 birds. To collect the samples, we watched a flock of great knots from a distance then collected samples into sterile collection cups once the flock had moved elsewhere. For the post-migration sample, we confirmed with a local shorebird counter that the knots had returned in large numbers (nearly 2000 in the flock, > 90% of them great knots) and were still showing signs of reduced body weight following their long-distance migration. In the field, faecal samples consisted of five aggregated great knot faeces, identifying them based on their characteristic cylindrical shape (*pers. comm.* Amanda Lilleyman). Similarly, each sand sample contained five small scoops of sand from where the birds had been located across a small area similar to that encompassed by each group of bird faeces. Pipis were collected by sieving sand near the waterline.

### Amplicon Sequencing and Analyses

Samples were stored at − 80 °C until DNA extraction with the Qiagen DNeasy PowerSoil Pro Kit (Qiagen, Valencia, USA; cat 47014). We extracted DNA from approximately 0.3 g of sand and faecal samples (sand: 0.332 ± 0.0535; faeces: 0.328 ± 0.0601) and five pipis per extracted sample (0.170 ± 0.0427 g). Pipis were rinsed three times with ultrapure water before being crushed with forceps into the PowerBead Pro tubes for extraction. We amplified the V4 region of bacterial 16S rRNA gene with the 515F/806R primers [18, 19] to characterise the whole bacterial microbiome. Because 16S rRNA gene amplicon sequencing does not allow for identification of *Vibrio* reads to species level, we used a portion of the *Vibrio* hsp60 gene as a second locus to identify *Vibrio* species in the communities [20]. Amplicons were sequenced using a 2 × 250 paired-end strategy on an Illumina NovaSeq 6000 platform at Ramaciotti Centre for Genomics, Sydney on a run with other samples. In addition to the samples, we extracted DNA and conducted PCRs on two extraction controls, which did not amplify and we did not include in the sequencing run.

Demultiplexed paired-end reads of 16S rRNA gene sequences were denoised with dada2 in QIIME2 2022.8 [21, 22] with default parameters, assigning ASVs to the Silva v138 database with the sklearn classifier [23–25]. ASVs unassigned (i.e., non-bacteria) or identified as chloroplast or mitochondria were removed. After subsetting the project’s samples, we removed ASVs present in only one sample or representing less than 0.01% of the reads.

Due to varying read depths among the samples, we used alpha-rarefaction (*n* = 10 repeats) to calculate richness as an average per sample with 160 reads. Similarly, we used beta-rarefaction with 160 reads per sample to perform Bray–Curtis principal coordinates analysis. We ran analyses in RStudio v2022.12.0 with R v4.2.1 [26, 27]. Alpha diversity was analyzed with ANOVA and Tukey’s HSD post-hoc test, testing the impact of sample type (faeces, pipi, sand), sampling season (March, September), and their interaction. We analyzed beta diversity on 160 reads per sample with adonis2 and betadisper and in the vegan package [28]. Lastly, we used the phyloseq package [29] to visualise sample connectivity in network form.

To identify *Vibrio* spp. in the samples, demultiplexed forward reads of hsp60 gene sequences were denoised with dada2 as above, truncating at 240 bases and trimming the first 4 leading bases, with otherwise default parameters. Resultant ASVs were aligned to a *Vibrio* spp. database [20] with BLAST+ [30] and 90% identity classification, removing unassigned ASVs from the dataset. The remaining *Vibrio* spp. ASVs were assigned to taxa with the sklearn classification [23]. Because only three faecal samples had *Vibrio* reads, we limited our discussion of these data to qualitative comparisons. After inspecting rarefaction curves, the dataset was rarefied to 270 reads per sample to assess species barplots, principal coordinates analysis, and network analysis. Amplicon sequence data have been submitted to NCBI’s Sequence Read Archive (BioProject ID: PRJNA1021386).

## Results and Discussion

### Limited Connectivity in Whole Bacterial Microbiomes

**Fig. 1:**
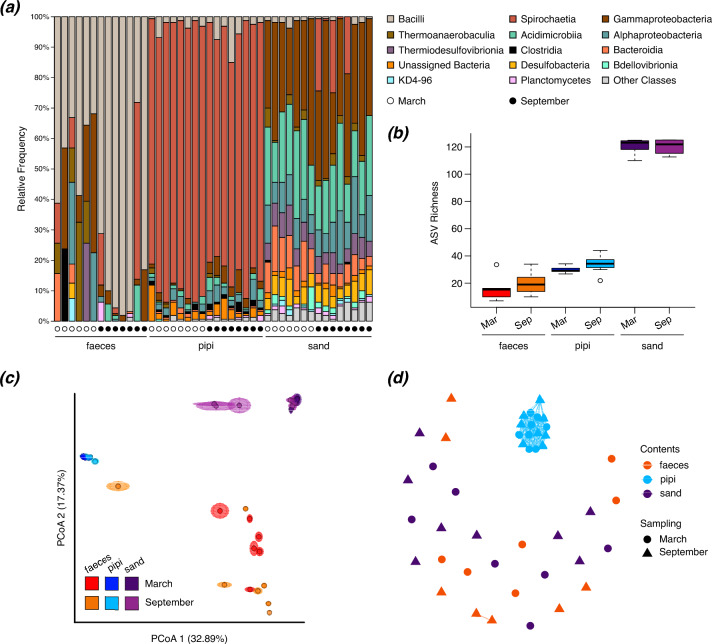
Bacterial communities differed among sample types but not between sampling periods. **a** Barplots of the major bacterial classes in field samples, including classes accounting for > 3% of the relative abundance in any sample. **b** ASV richness was greatest in sand samples and lowest in faecal samples (Tukey’s HSD, *P* < 0.0001 each). **c** Bray–Curtis principal coordinates analysis revealed no clustering in faecal samples and strong clustering in the other sample types. **d** Bray–Curtis network (maximum distance = 0.4) supports minimal connectivity of great knot faecal samples with their food or the environment. Eight samples were collected per sample type per sampling period, though three faecal samples and one sand sample failed during library prep and sequencing

Great knot faecal samples were largely comprised of Bacilli, particularly Mycoplasmataceae and Erysipelotrichaceae, while communities in pipis were dominated by spirochaetes, and sand samples had more evenly diverse communities with many bacterial classes (Fig. 1a). Among other birds, Firmicutes and Proteobacteria [3, 7] are the dominant bacterial phyla in gut communities. Both phyla had high relative abundance in our pre-migration samples, though Firmicutes (Bacilli) replaced other taxa after migration.

We found differences among the sample types in both alpha and beta diversity metrics. In alpha diversity, sand samples had significantly greater richness than the other two sample types, while faeces had the lowest richness (Fig. 1b; Contents: *F*_*2,38*_ = 1080.56, *P* < 0.0001). Beta diversity also differed among the sample content types (Fig. 1c; Contents: *F* = 17.83, *R*^2^ = 0.46, *P* = 0.001), though pipi samples had differing beta dispersion from the other sample types. Pipi samples clustered tightly in principal coordinates space, while faecal samples were spread quite broadly. In faecal samples, low richness could be due to low resident biomass, with faecal microbes mainly associated with foodborne transient taxa, while dispersion of the faecal samples in principal coordinates space supports previous results of low phylogenetic signal in bird gut communities ([6], but see [9]). Lewis et al. [10] found that changing environment and food source can quickly alter gut microbes of passerines, and Dion-Phénix et al. [11] also found overlap in gut microbes in blue tits and their available food source. However, had the great knots’ faecal bacteria been strongly affected by their food source in this study, we would expect connectivity between sample types in network analysis. In our samples, only the pipis showed connectivity in network analysis (Fig. 1d).

In comparisons between the two sampling time points, we found similar richness (Sampling: *F*_*1,38*_ = 1.697, *P* = 0.2; Contents × Sampling: *F*_*2,38*_ = 0.514, *P* = 0.6) and beta diversity (Fig. 1c; Sampling: *F* = 1.63, *R*^2^ = 0.02, *P* = 0.09; Contents × Sampling: *F* = 1.42, *R*^2^ = 0.04, *P* = 0.1) across time. The lack of a seasonal component suggests that the communities are relatively stable across time, especially in pipis, which have strong connectivity in network analysis. In the birds, statistical similarity over time may be a consequence of their quick digestion and low microbial retention, resulting in varied communities among individuals in general. Studies of other migratory birds have found that, though bird gut microbiomes differ between breeding and overwintering ranges, the communities can shift within the first day of returning to a site [10, 31]. Consequently, apart from migratory birds temporarily retaining key bacterial taxa perhaps linked to energy storage, their gut communities can become similar to resident conspecifics within a few days of returning from migration [32]. We might expect that local food source should drive such rapid community changes, but the absence of an overlap between the bird and pipi samples suggests that not just dietary shifts, but other concurrent factors (e.g., physiological and environmental) may also contribute to these shifts.

Regarding the initial hypothesis of greater connectivity before than after migration, we found that relaxing the maximum distance for the network analysis actually showed stronger grouping among the post-migration samples. Along with the relative increase in Bacilli (or decrease in other microbes) after migration, these results could be because physiological stress due to migration had altered the gut bacteria. Exercise and stress can affect gut microbiomes [33] and the birds sampled in September 2022 had recently flown several thousand kilometers, as indicated by their visibly poorer body condition. Our results suggest that migration status and low microbial specificity greatly affected the great knot gut microbiomes represented in the faecal samples collected after the birds returned.

### Presence of Vibrios but no Connectivity Among Sample Types

**Fig. 2:**
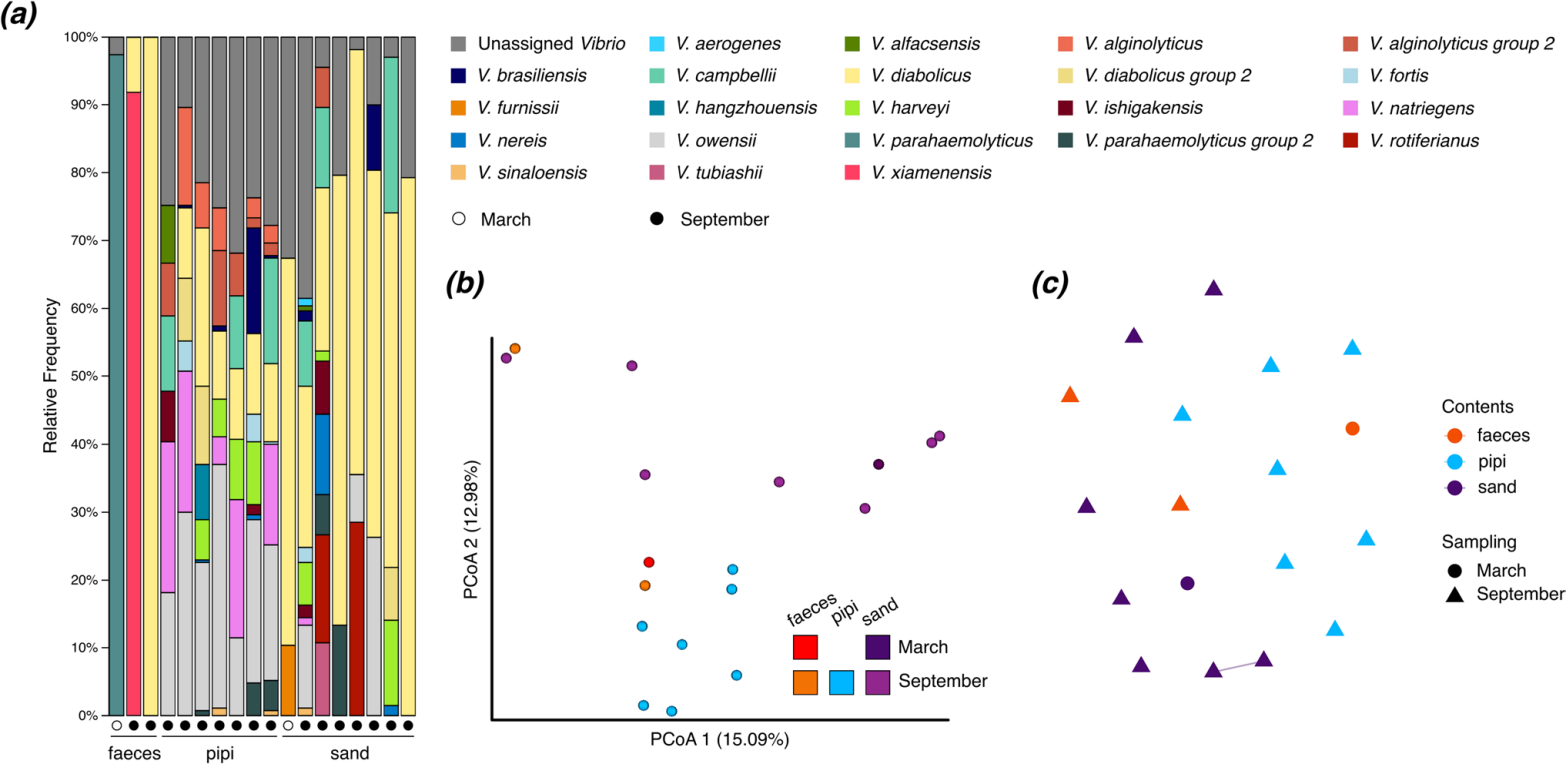
Faeces, pipis, and sand have diverse *Vibrio* species with minimal community connectivity. **a** Barplots of *Vibrio* spp. **b** Bray–Curtis principal coordinates analysis. **c** Bray–Curtis network analysis (maximum distance = 0.4)

All three sample types contained potentially pathogenic *Vibrio* spp., including *V. campbellii* and *V. owensii* (emerging aquatic animal pathogens), and *V. parahaemolyticus* (a human pathogen) (Fig. 2). Pipi and sand samples diverged in principal coordinates space, but we found minimal connectivity within the sample types, and no connectivity in *Vibrio* communities between sample types. Interestingly, the three bird samples with detectable *Vibrio* each had a single different dominant species, which could be a sign of one species outcompeting the others in the gut, or differential colonisation success. One bird sample was dominated by *V. xiamenensis*, which was not detected in either sand or pipis.

Though the data presented here stem from DNA samples, in a pilot study (February 2022) we cultured samples on two *Vibrio* growth media, confirming that great knot faeces (as well as the two other sample types) contain living *Vibrio* spp. (*unpublished data*). Elsewhere, pathogenic *V. parahaemolyticus* has been cultured from faecal samples of other waterbirds [34]. As migratory birds are implicated in the spread of multiple human pathogens by other means (e.g., as biological carriers through infection, as transport of pathogenic spores or pathogen-carrying ectoparasites; [2]), their capacity to spread bacteria, including potential pathogens, via their gastrointestinal tract warrants further research. In particular, we hypothesise that animals may act as vectors for *Vibrio* movement via trophic interactions and migration in addition to the known dispersal route of warm currents.

The identification of *Vibrio* spp. in this study was based on a sequence database from 2019 [20]. The high relative abundance of unassigned *Vibrio* ASVs highlights the limitations of our knowledge of the genus. Most of the unassigned reads were found in the pipi samples (Fig. 2a), revealing tropical invertebrates and their bacterial symbionts as an understudied system deserving more focus.

## Conclusion

Migratory shorebirds annually move thousands of kilometers, interacting with many systems and environments along the way. Despite interactions with varied food sources, however, gut bacterial communities appear to reflect recent meals, suggesting that few bacterial taxa are able to colonise the bird gut in the long-term. Our samples of great knot faeces, their bivalve prey, and nearby sand provided results contrary to our expectations regarding the impacts of dietary bacteria on gut communities. We found negligible similarities between faecal and pipi bivalve communities, and no shift in community connectivity before and after the birds’ migration. As expected, however, we did detect a relatively rich *Vibrio* community in the samples, with bird samples dominated by one or few taxa. This dominance may indicate colonisation in the bird guts, supporting the potential for migratory great knots to spread gut bacteria over long distances. Our data here were limited by low bacterial reads in faecal samples, possibly associated with low gut bacterial biomass. Further studies using source tracking would help identify the extent of influence that migratory birds have on bacterial movements.

## Data Availability

Amplicon sequence data have been submitted to NCBI’s Sequence Read Archive (BioProject ID: PRJNA1021386).
